# Software Tool for the Prosthetic Foot Modeling and Stiffness Optimization

**DOI:** 10.1155/2012/421796

**Published:** 2012-03-29

**Authors:** Matija Štrbac, Dejan B. Popović

**Affiliations:** ^1^Faculty of Electrical Engineering, University of Belgrade, 11000 Belgrade, Serbia; ^2^Aalborg University, Center for Sensory Motor Interaction, 9200 Aalborg, Denmark

## Abstract

We present the procedure for the optimization of the stiffness of the prosthetic foot. The procedure allows the selection of the elements of the foot and the materials used for the design. The procedure is based on the optimization where the cost function is the minimization of the difference between the knee joint torques of healthy walking and the walking with the transfemural prosthesis. We present a simulation environment that allows the user to interactively vary the foot geometry and track the changes in the knee torque that arise from these adjustments. The software allows the estimation of the optimal prosthetic foot elasticity and geometry. We show that altering model attributes such as the length of the elastic foot segment or its elasticity leads to significant changes in the estimated knee torque required for a given trajectory.

## 1. Introduction

**Figure 1:**
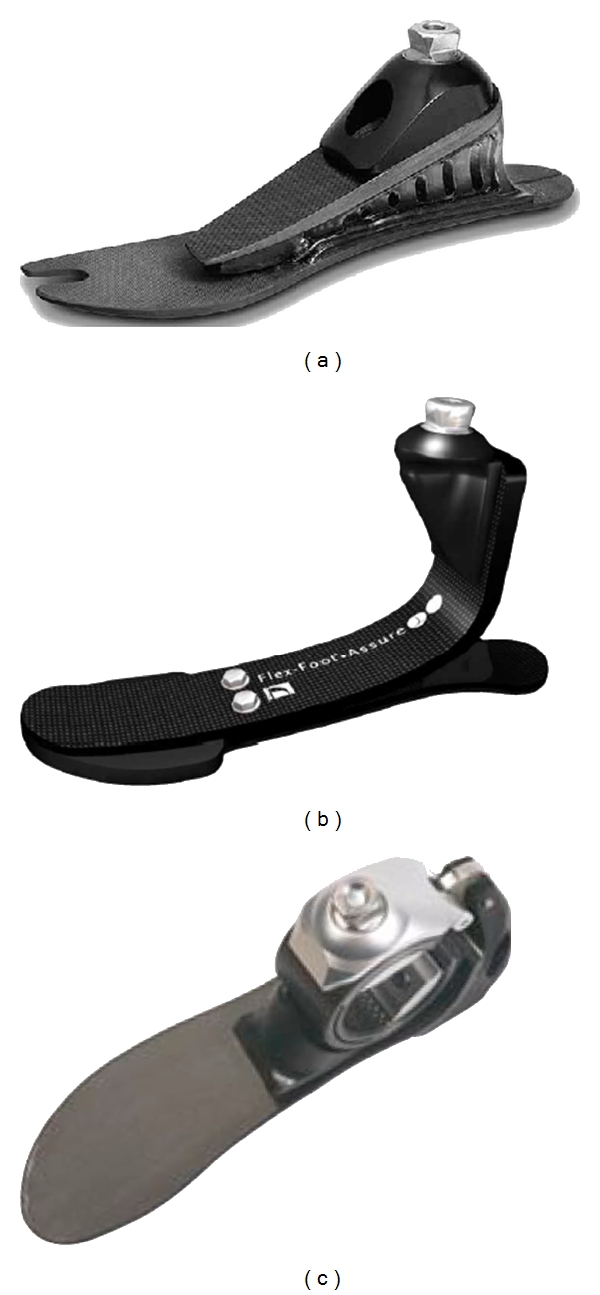
Flex-Foot Axia, Ossur, Island (a), Flex-Foot Assure, Ossur, Island (b), and 1C40 C-Walk, Otto Bock Germany (c). Adapted from [17, 18].

Artificial feet (Figure 1) currently often used within transfemural and transtibial prostheses comprise elements for heel impact absorption and energy accumulation during the loading of the leg. These features are instrumental for comfortable and energy-efficient gait [1]. The mechanics of the C-foot and various versions of the flexfoot-based feet follow the principles described in several patents [2–6].

**Figure 2:**
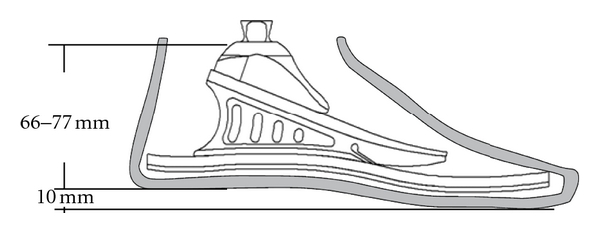
The sketch of the model of the Flex-Foot Axia, Össur, Island. Adapted from [17].

The sketch of the foot (Figure 2) shows that the sole of the foot is elastic at the rear and front ends, while the area under the ankle joint is rigid.

In this paper, we use a biomechanical model that considers the elastic sole. The model that we adopted takes into account the longitudinal bending of the foot and carries over the kinematical and kinetic changes to other joints as has been done in previous studies [7]. We present here the procedure that allows the design of the leg based on the individual characteristics of the patient and the gait modality of interest.

**Figure 3:**
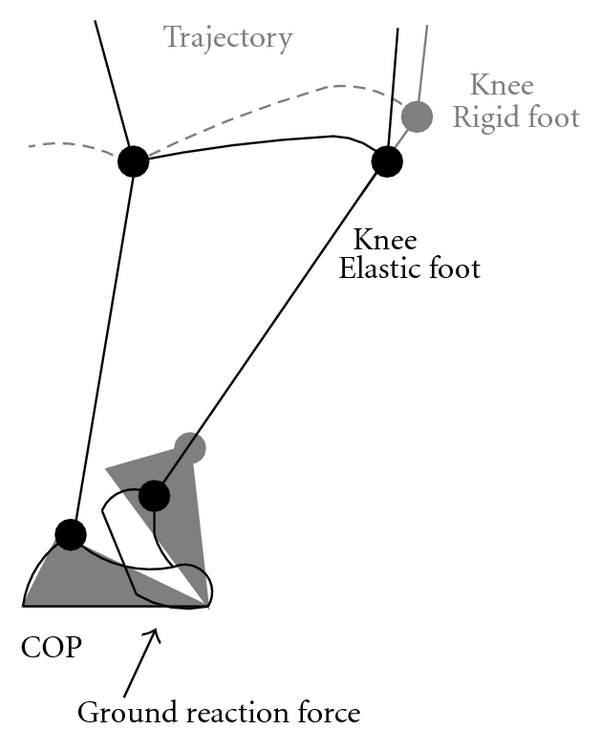
Trajectory of the knee joint for the rigid and elastic foot.

The sketch shows the geometrical differences in the knee joint position during the gait (Figure 3) when the model with the flexible foot is used. The altered geometry results with the difference in the distance between the knee joint and the point (COP) where the ground reaction force acts. This change results with the alteration of the knee joint torque that is required [8, 9] for the tracking of the desired trajectory. We suggest that the flexible bending model of the foot yields to reduced knee joint torques, ultimately reduces stress on the muscle activities of the knee extensors during the stance phase of the gait. In short, the goal of this study was to assist the individual design procedure of the prosthetic foot for the given user and the selected gait activity.

Human foot motion during stance can be described as a smooth progression between four phases: (1) heel strike, (2) flat foot, (3) heal lift, and (4) toe push-off. The model and proposed optimization methods are focused on the characteristics of the forefoot since the push-off phase and heel lift require the most energy during the gait cycle.

In order to justify the reasons for this research, we review shortly the state of the art that dominated recent literature. New designs and materials in prosthetic foot-ankle assemblies offer the individual with lower limb amputation wide choice among the prostheses with rather similar characteristic, which complicates the task of the prosthetic clinic team in formulating the most appropriate prescription for each patient. The most important clinical distinction is related to the internal design characteristics, which enable the component to simulate some actions of the human foot [10]. Over the last 20 years, many studies concentrated on comparing various properties of these assemblies [11–14] and came to conclusion that the optimal design choice depends on the individual walking style. However, the most popular prosthetic devices today (Carbon Copy II, Otto Bock SASH, Greissinger foot, SAFE foot, Seattle Natural, Flex-Foot) all consist of the (1) elastic keel which provides the energy transfer from the heel strike through to the toe off and the dorsiflexion required for natural ambulation and (2) the considerably stiff heel, which provides the impact absorption at heel strike and the kinetic energy required for a smooth transition between the heel strike and the toe off [1]. Our model incorporates these two elements and allows us to mathematically optimize their stiffness based on a given walking pattern for any geometry of the prosthetic foot.

This presentation concentrates on the optimization procedure. Thus, for the geometry in question, it is particularly important to find the upper bound of elasticity above which the foot becomes rigid and the lower bound below which the elasticity of the foot will result in a change in knee torque. In other words, we estimate the limits on the elasticity of a foot to match the patterns of healthy feet. The simulation uses gait and anthropometric data from healthy individuals. We present a software tool that can minimize knee torque based on biometric subject data.

The results of this paper are relevant to the design of optimal geometry and stiffness identification for prosthetic feet. This study does not consider the powering of the ankle joint that has recently been introduced (e.g., PowerFoot One iWalk, Cambridge, MA or Proprio Foot Ossur, Reykjavik, Iceland [15]).

## 2. Methods

**Figure 4:**
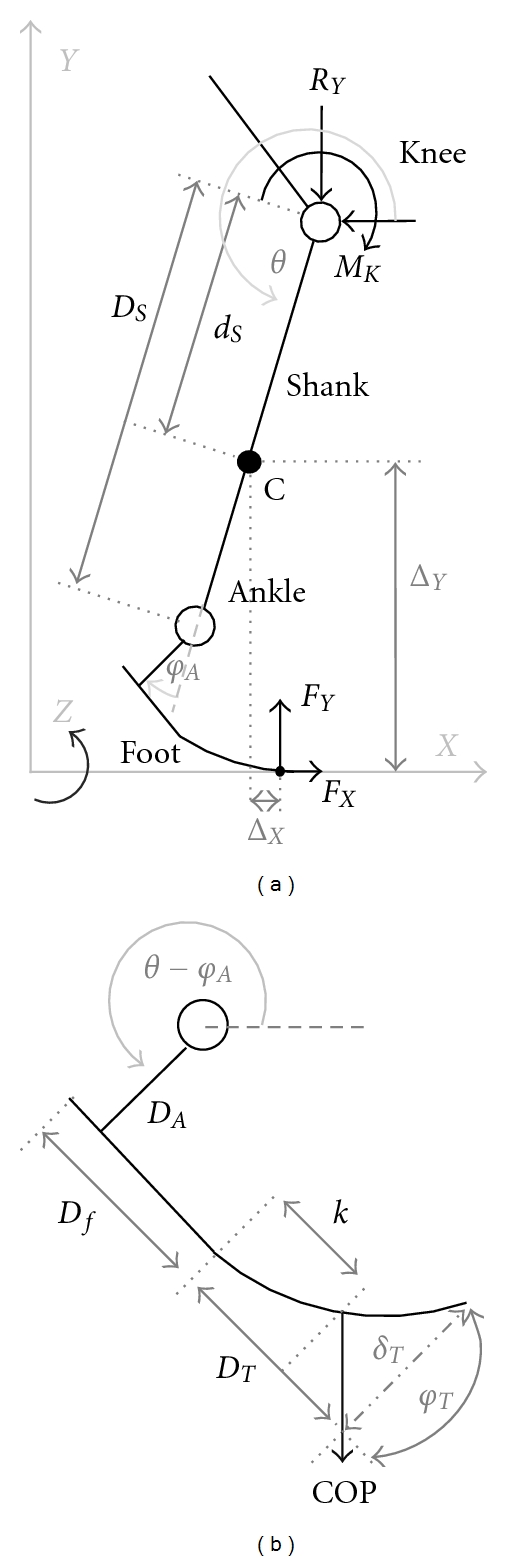
The model of the leg (a) and the foot (b) with the annotations used in the mathematical model.

The mathematical model for inverse dynamics analysis of human gait is a set of equations based on the laws of momentum and kinetic momentum. The model presented is planar with only one active degree of freedom (DOF) at the knee joint and a bending foot that alters its geometry according to the load experienced along its length (Figure 4). The ankle joint motion was introduced since the obtained data are from the walking of the healthy individual; yet, we are not considering the power at this joint. This simplification was recommended in order to form a model that could produce the healthy human gait pattern. The list of notations is presented in the form of a table (Table 1).

If we assume the leg below the knee joint as one segment (no active joints), the kinetics is defined by three second-order equations, however, with the flexible geometry that introduces the ankle rotation:

(1) $$\begin{array}{c} m{\ddot{x}}_{C}=F_{x}-R_{x}, \end{array}$$

(2) $$\begin{array}{c} m{\ddot{y}}_{C}=F_{y}-R_{y}-mg, \end{array}$$

(3) $$\begin{array}{c} J_{Cz}\ddot{\theta }=F_{x}\Delta_{y}-F_{y}\Delta_{x}+R_{x}D_{S}\cos\theta -R_{y}D_{S}\sin\theta -M_{K}. \end{array}$$

From (1) and (2), we can eliminate the knee driving forces in (3) and express the knee torque as a function of the kinematical data and the external, ground reaction forces:

(4) $$\begin{array}{cc} M_{K} & =\left(F_{X}-m{\ddot{x}}_{C}\right)D_{S}\cos\theta -\left(F_{Y}-m{\ddot{y}}_{C}-mg\right)D_{S}\sin\theta \\ & \;-F_{Y}\Delta_{x}+F_{X}\Delta_{y}-J_{Cz}\ddot{\theta }. \end{array}$$

The segment kinematics need to be estimated. In our case, this was done by a stereometric system of cameras. The ground reaction forces have been estimated using the force platform. The parameters required for the optimization are also the horizontal and vertical distances from the center of mass (CM) and the position of the ground reaction force (COP) Δ_*x*_ and Δ_*y*_.

The foot model was designed to include one elastic beam component that replaces the flexion of the metatarsal joint (*D*_*T*_) and two rigid components that imitate the unyielding central portion of the human foot (*D*_*f*_) and externally controlled vertical part of the foot (*D*_*A*_). This model allows that the optimization uses the parameters of these three segments (geometry and elasticity). This allowed us to study the influence of the term *F*_*y*_Δ_*x*_ + *F*_*x*_Δ_*y*_, which describes the moment acting at the center of mass.

The moment arm can be determined based on trigonometric formulas if the toe bending parameters represent the amount and angle of beam deflection:

(5) $$\begin{array}{cc} \Delta_{x} & =-\left(D_{S}-d_{S}\right)\sin\theta -D_{A}\sin\left(\theta -\varphi_{A}\right)\\ & \;+\left(D_{f}+D_{T}\right)\cos\left(\theta -\varphi_{A}\right)+\delta_{T}\sin\left(\theta -\varphi_{A}\right),\\ \Delta_{y} & =-\left(D_{S}-d_{S}\right)\cos\theta -D_{A}\cos\left(\theta -\varphi_{A}\right)\\ & \;-\left(D_{f}+D_{T}\right)\sin\left(\theta -\varphi_{A}\right)+\delta_{T}\cos\left(\theta -\varphi_{A}\right). \end{array}$$

These formulas follow the leg and foot models shown in Figure 4.

According to Euler-Bernoulli beam theory, beam deflection and the angle of deflection in a simple cantilever beam can be described using the two following equations:

(6) $$\begin{array}{c} \delta_{\text{deflect}}=\frac{Pk^{2}}{6EI}\left(3L-k\right), \end{array}$$

(7) $$\begin{array}{c} \varphi_{\text{deflect}}=\frac{Pk^{2}}{2EI}, \end{array}$$

where *P* is the force perpendicular to the beam and exerted at the distance *k* along the length of the beam *L*. This is equivalent to a GRF being exerted on the foot at some distance *k* along the length of elastic segment. Hence, this force can be easily estimated:

(8) $$P=\left|F_{x}\sin\left(\theta -\varphi_{A}\right)+F_{y}\cos\left(\theta -\varphi_{A}\right)\right|.$$

Since the foot is decomposed into simple beam structures, these equations are appropriate for the representation of geometrical variation required to calculate Δ_*x*_ and Δ_*y*_ during foot bending.

In the presented foot model, there are three geometrical parameters that can be altered to optimize a prosthesis: the length of the bending segment (*D*_*T*_) and the lengths of the rigid structures (*D*_*A*_, *D*_*f*_). If these parameters are defined, based on (6) and (7), the only parameter left to alter is *EI*, which represents the stiffness of the beam. The cross-sectional moment of area *I* can be estimated based on the shape and geometry of the prosthetic foot of interest. In general, this moment varies throughout the foot because its geometry is not uniform. Thus, this value should be calculated as a length-dependent variable. In this presentation, we selected the cross-sectional moment of inertia  *I* = 1.8 · 10^−7^ m^4^. This value was selected as the typical value for some of the commercially available devices.

Here we concentrate on estimating the elasticity of the foot *E* for the selected value of the cross-sectional moment of inertia. However, this can be easily changed toward the analysis of the product *IE*; hence the modified cross-sectional moment of inertia form the one presented here.

The software presented has been tested for various gait modalities. In this paper, we present only one dataset. These data come from the gait analysis of a female subject walking in a gait laboratory (Movement Analysis Laboratory, Rizzoli Orthopedic Institute, Bologna, Italy) and include measurements of body segment kinematics based on the CAST protocol [16]. The recordings include the ground reaction force estimates made using the Kistler force platform.

### 2.1. Optimization Software

A software tool for prosthetic foot analyses was developed using an interactive MATLAB-based application with a graphical user interface. The program initially shows the subject's anthropometric parameters, which will later define the segmental inertial properties at play (i.e., segment mass, the center of gravity, and the moment of inertia). For these calculations, we used simple geometric modeling combined with the measured data in the same way as introduced in [19], bearing in mind that for the physically disabled, this process will be slightly different [20].

**Figure 5:**
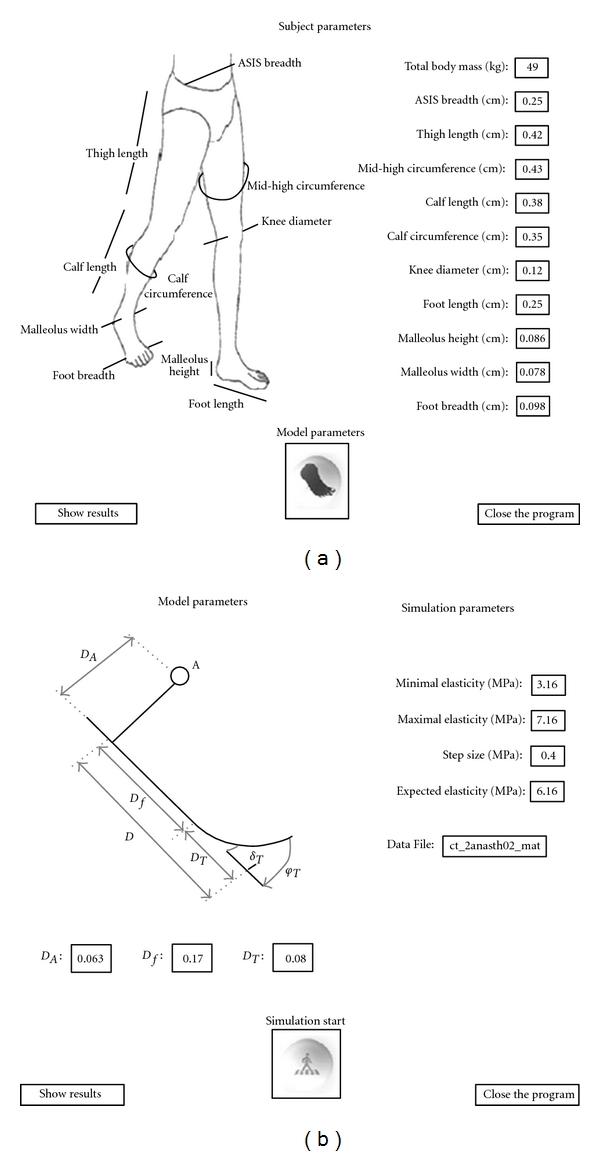
The graphical interface that defines inertial properties using the anthropometric data of the subject (a) and the graphical interface that defines the model's geometrical characteristics and simulation properties (b).

The anthropometric parameters shown in Figure 5 are the actual values for the real subject used in the simulation presented. The parameters need to be set before simulation to correspond to the subject whose gait is being analyzed. The button in the center of the screen will direct the user to the second screen, where he can determine the geometric characteristics of the prosthetic foot model and some of the simulation properties. The user can leave the program and the simulation at any time simply by clicking the button at the far right of the screen.

The second screen is similar to the first one, with a button in the center that starts the simulation. This screen also includes the fields for entering parameters that define the foot model and the fields determining the simulation process. The foot model parameters that need to be entered are dimensions of the one bending component *D*_*T*_ and two rigid segments *D*_*f*_ and *D*_*A*_, as previously described. The lengths can be set, but the aggregate foot length (total length) must remain the same. The simulation parameters include the initial prosthetic foot elasticity and the optimization parameters. These parameters consist of the lower and upper bounds of elasticity and step size for the iterative search for the optimal modulus of elasticity. Initial elasticity is used only for the comparison of the final results where it will be presented in respect to the rigid foot model and the model with optimal elasticity, while the other parameters will have influence on the simulation as well as the final result. The last field on this screen contains the name of the file where the kinematical and dynamic data are stored. This data need to include hip, knee, and ankle join center kinematics in sagittal plane, ground reaction forces, and the positions of those forces (i.e., COP).

**Figure 6:**
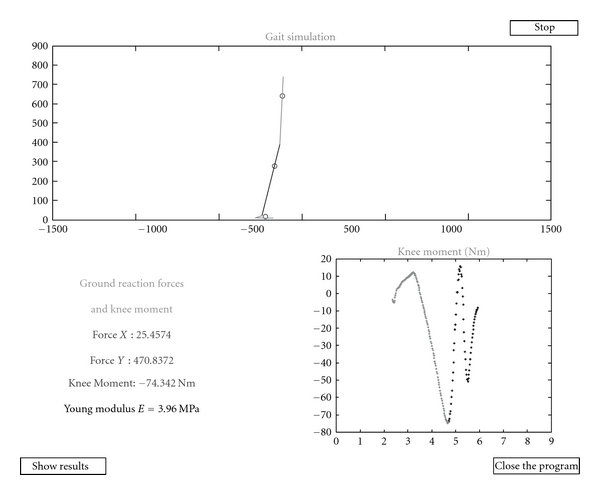
Simulation window and the values of interest in the captured instance of time.

The simulation screen includes two graphs and four text fields that track the changes in the parameters of interest in every sample of time (see Figure 6). Also, the center of mass is indicated for every segment with a circle. The figure in the upper half of the window shows the model position in the sagittal plane. The lower right part of the screen shows a graph that indicates the knee torque in the presented instance. Text fields to the left capture the vertical and the horizontal components of the ground reaction force, the knee torque in the presented time sample, and the modulus of elasticity in the current iteration. As previously mentioned, this simulation routine will be conducted at every step for the elasticity module within the range of the elasticity bounds defined in the simulation properties. This simulation protocol is intended to give the user the ability to monitor the effect of the model changes in the time domain and to identify the fragments of the gait cycle that the possible instabilities or peaks in the knee torque create. This insight into gait phase and model feature dependency thus helps to generate better model parameter allocation and better model formation.

Finally, for ease of understanding, the input variables and the optimized knee torque are presented as a function of time. The input variables include joint center kinematics in the vertical and horizontal directions and the vertical and horizontal components of the ground reaction force. They are presented in line with the simulation results for better anticipation of the correlation. The output from the simulation includes three differently calculated knee torque signals. Two of them present different elasticity levels in the bending foot model, the optimal elasticity and the user-defined expected elasticity. The expected elasticity model is included in this software for three reasons: (1) as a reminder that the optimal knee moment does not always represent most human foot prostheses; (2) the optimization bounds should be set based on (6) and (7), and (3) to provide explicit predictions regarding different levels of model elasticity associated with the knee moment. The knee torque for the stiff foot is presented as the upper boundary of the knee torque in the elastic model. If the model knee torque exceeds this bound, then the lengths of the elastic segments must be changed.

## 3. Results

**Figure 7:**
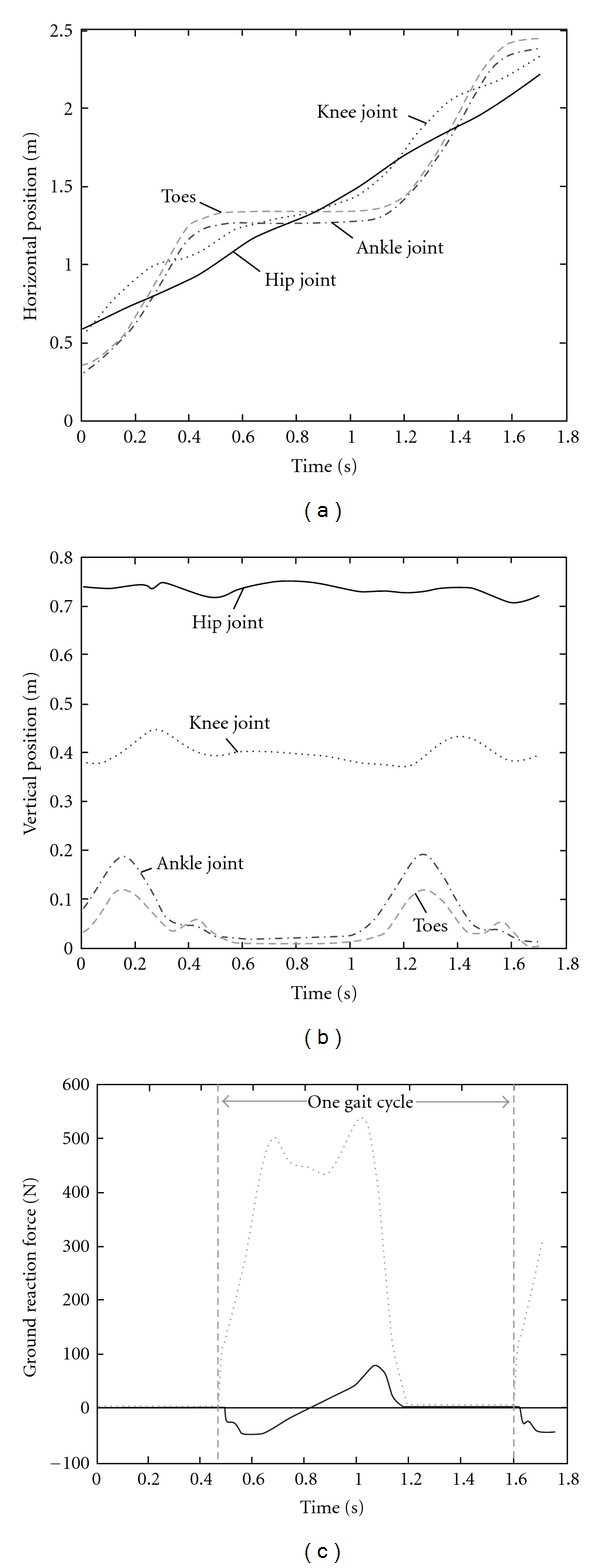
The kinematics and ground reaction forces used for simulation. The subject was a young female with body mass of 52 kg, height 1.64 m. The gait cycle lasted *T* = 1.14 s, and the stride length was 1.4 m (*v* = 1.22 m/s).

The input data required for the simulation are presented in Figure 7. The horizontal and vertical positions were used to estimate the joint angles required for the simulation. The center of pressure was estimated from the data recorded by the force platform.

We assumed that the maximal angle between the foot and the walking surface is approximately 30 degrees. Using the information about the maximum ground reaction force, we calculated (6) that the elasticity of a healthy foot is approximately 6.16 MPa. Based on this value, we chose an elasticity range between 4.16 and 8.16 MPa. We also set the upper and lower limits for maximum foot bending.

**Figure 8:**
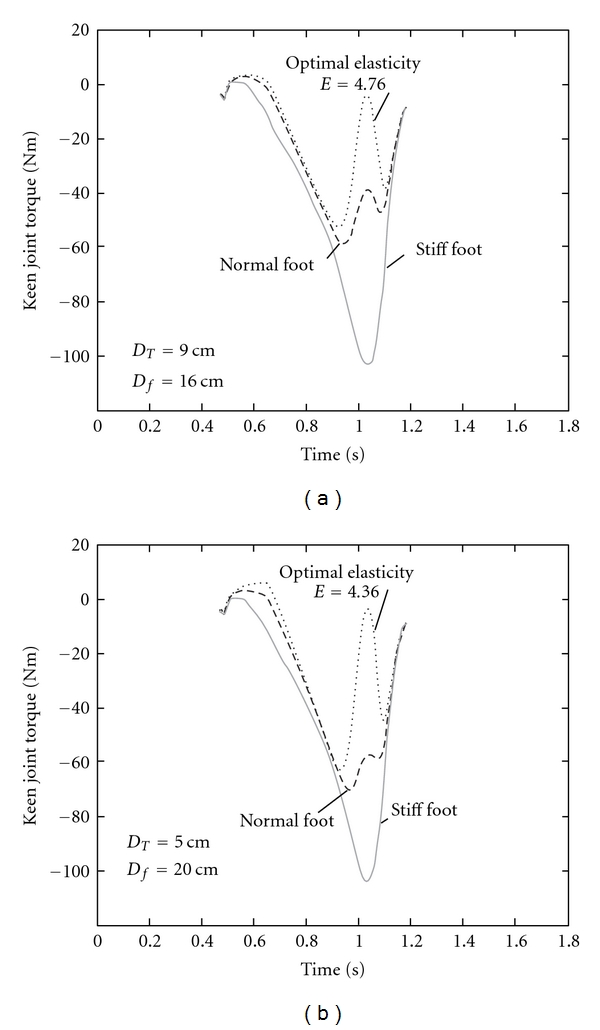
The knee joint torque calculated for a normal foot, a foot with optimal elasticity determined using the software presented in this study, and a stiff foot (*D*_*T*_ + *D*_*f*_ = 25 cm). (a) illustrates the case featuring a shorter bending segment (*D*_*T*_ = 5 cm), and (b) illustrates the case featuring a longer bending segment (*D*_*T*_ = 9 cm).

The software makes it possible to select any ratio between the lengths of the bending and stiff segments of the foot. The software estimates the optimal stiffness based on the minimal knee joint torque. From the results shown in Figure 8, it is easy to conclude that the elastic foot model produces lower knee joint torque than the stiff foot model (*≈*50%) and that the increase in the ratio between the bending and stiff prosthetic segments improved this figure even more (*≈*10 Nm). The optimal elasticity in the first case was *E* = 4.36 MPa (bending/stiff = 1 : 4); in the second case, it was 4.76 MPa (bending/stiff = 9 : 16).

## 4. Discussion

This paper presents the software developed for use in designing the optimal foot for prosthetic applications. The software also allows the selection of the appropriate motor unit if the prosthesis is to be externally driven. This is possible because the software determines the joint torque range required to track the desired trajectory and the dynamics that it needs to guaranty.

The software makes it possible to test the performance for various gait modalities and set the parameters to appropriate values for the amputee who would eventually use the transfemoral prosthesis.

We demonstrated that it is important to select the appropriate stiffness and geometry of the prosthetic foot to minimize the power needs at the knee joint. The shape of the knee joint torque will resemble the pattern of normal walking; thus, the joint torque at the hip (volitional control by the amputee) is likely to be almost normal.

The software also simulates walking on various terrains (on sloped ground, up the stairs, etc.), which is of great interest for multimodal control [21–23].

This model does not take into account the elastic deformation of the heel during initial ground contact or the impact on the heel during heel contact. These factors could be incorporated by including another bending beam on the back of the rigid segment. The elasticity and geometry of this beam could be analyzed using the methods presented here.

## Figures and Tables

**Table 1:** List of notations.

Symbol	Definition
${\ddot{x}}_{C}$	Horizontal acceleration of the leg
${\ddot{y}}_{C}$	Vertical acceleration of the leg
COP	Position of the center of pressure
*D*_*A*_	Length of the ankle segment
*D*_*f*_	Length of the unyielding foot segment
*D*_*T*_	Length of the toes segment
*d*_*S*_	Distance from the knee to the center of mass
*D*_*S*_	Length of the shank
Δ_*x*_	Horizontal distance from the CM to COP
Δ_*y*_	Vertical distance from the CM to COP
*F*_*x*_	Horizontal ground reaction force
*F*_*y*_	Vertical ground reaction force
*δ*_*T*_	Amount of deflection in the elastic beam
*φ*_*T*_	Angle of deflection in the elastic beam
*φ*_*A*_	Externally controlled ankle joint angle
*EI*	Bending stiffness of an elastic segment
*J*_*Cz*_	Moment of inertia for the leg and foot
*m*	Mass of the leg and foot
*θ*	Angle of the shank with respect to the horizontal
*M*_*K*_	Joint torque at the knee joint
